# American Veterinary College (New York)

**Published:** 1890-09

**Authors:** 


### VETERINARY COLLEGE NOTES.

*American Veterinary College (New York).*—The American Veterinary College has issued its annual announcement, showing many important and advantageous changes and additions. Dr. R. S. Huidekoper has been appointed Professor of Sanitary Medicine and Veterinary Jurisprudence; Dr. James E. Ryder, Professor of Obstetrics and Canine Pathology; Dr. R. K. Bell, Professor of Materia Medica and Therapeutics. A Quiz Faculty has been organized, and will be a useful addition to the teaching corps. It is expected that another year will see the college in a new building, which will offer unequaled facilities, and relieve the overcrowded lecture and laboratory rooms.

